# Torsion of intestinal parasitic myoma after laparoscopic morcellation: a case report

**DOI:** 10.1093/jscr/rjaa032

**Published:** 2020-03-17

**Authors:** Sofoudis Chrisostomos, Trouvas Dimitrios, Zioris Konstantinos

**Affiliations:** 1 Department of Obstetrics and Gynecology, Konstandopoulio General Hospital, Athens, Greece; 2 Mitera, Maternal Hospital, Athens, Greece

## Abstract

Uterine fibroids represent the most common type of benign tumor of female genital tract with rate of incidence between 20% and 30% in women older than 35 years. According to current bibliography, the percentage is still increasing due to the fact that many women are postponing their reproductive mapping. Laparoscopic morcellation of uterine fibroids and uterus specimen after hysterectomies has great clinical significance, concerning dissemination and implantation of uterine fragments inside the peritoneal cavity. Parasitic myomas reveal a rare entity, reflecting a broad spectrum of pathogenesis. In cases of parasitic uterine fibroids surgical dissection is mandatory to avoid signs of malignancy.

## INTRODUCTION

Uterine myomas consist of benign tumors originate from individual smooth muscle cells. They are mostly submucosal but can be intramural or subserosal and be at different locations of uterus [1,2].

They affect 20–30% of women older than 35 years [3].

Outside uterine cavity, parasitic myomas depict extremely rare, characterizing benign morphology, mimicking malignancy and setting difficulties in differential diagnosis with other tumors of gastrointestinal tract [4].

Pelvic or diffuse abdominal pain, menometrorraghia and pressure on urinary or intestinal tract reflect some of the most common symptoms.

They partially or completely separated from the uterus and receive their main blood supply from another source.

As possible mechanical explanation suggested the separation of pedunculated subserosal myomas, which may have outgrown blood supply.

Morcellation of uterine myomas, subtotal or total laparoscopic hysterectomy and following morcellation, can lead through dissemination of uterine segments inside peritoneal cavity into seeding of parasitic myomas [5].

More in detail, ‘Hormonal Replacement Therapy’ (HRT) using steroid hormones in cases of postmenopausal female patients can be hypothesized as dispositional factor concerning the development of parasitic myomas [6].

Overall incidence of parasitic myomas is estimated to be 0.2–1.25% with a median interval of 48 months from morcellation to diagnosis [7].

## CASE REPORT

We present a rare case of a 43-year-old female patient who was admitted at our hospital multiple times with episodes of abdominal pain and metrorrhagia.

Her past history includes loop electrosurgical excision procedure (LEEP), Hysteroscopic resection of endocervical polyp and a missed abortion.

Preoperative evaluation with transvaginal ultrasound revealed 7-cm intramural fibroid of the anterior uterine wall.

Patient underwent laparoscopic resection of intramural myoma followed by morcellation. Histopathology of specimen revealed uterine leiomyoma. The mentioned specimen removed in toto without remnants.

After a period of 6 months, patient had multiple episodes of diffuse abdominal pain and gastrointestinal disturbances with signs and symptoms of constipation.

Transvaginal ultrasound and pelvic magnetic resonance imaging revealed presence of parasitic myomas maximal diameter 9 cm, infiltrated inside the peritoneal cavity, infiltrating the gastrointestinal tract.

Patient underwent diagnostic laparoscopy for confirmation of diagnosis and treatment.

Upon inspection with laparoscopy, an enlarged torsioned with intestinal slings parasitic myoma was revealed. Of particular significance, presence of smooth muscle cells revealed large irregular vessels.

Two other parasitic myomas were visualized, adjacent to uterus in the peritoneal cavity.

Performed without injury to adjacent bowel followed by morcellation inside a contained bag and sent the specimen for histopathology.

**Figure 1:**
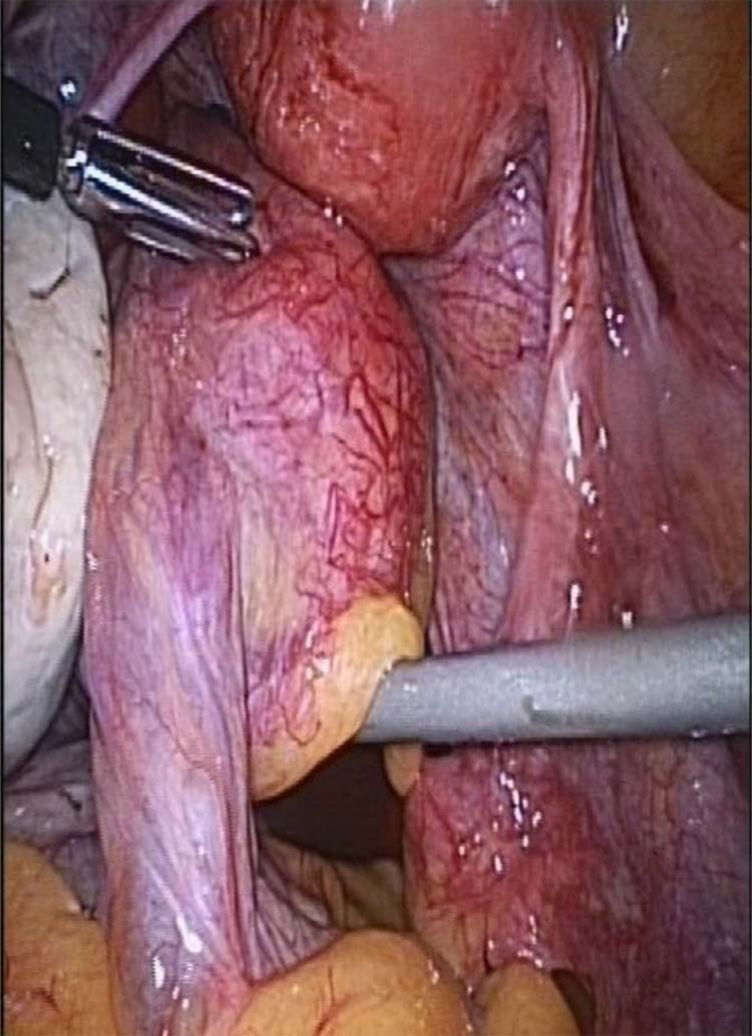
Torsion of intestinal parasitic myoma inside the peritoneal cavity.

**Figure 2:**
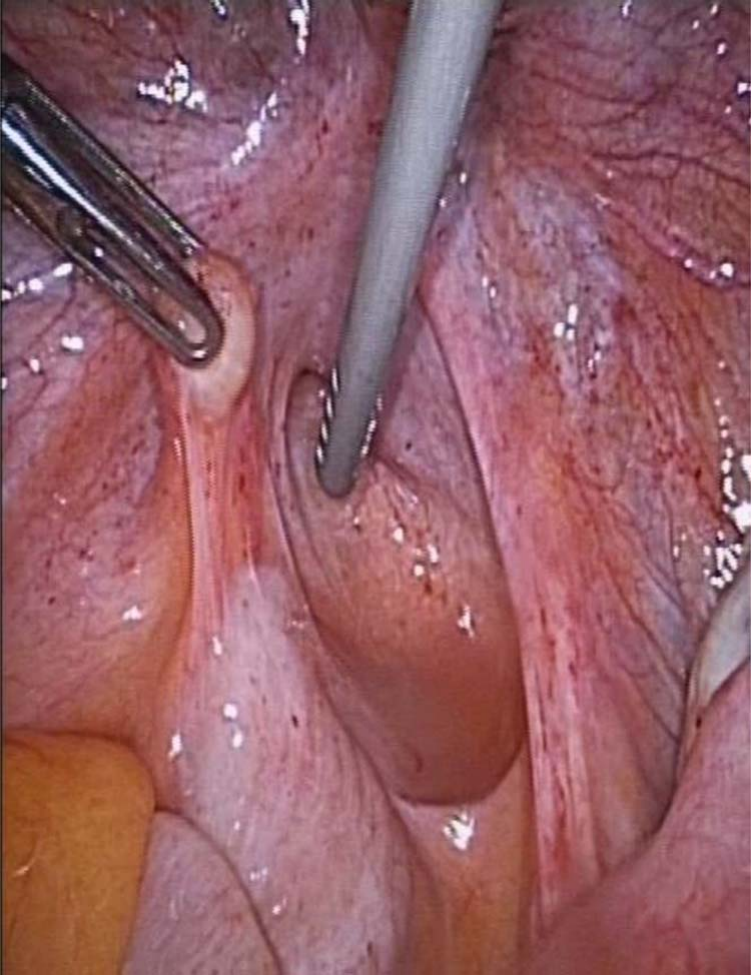
Parasitic myoma infiltrated inside the peritoneal cavity.

Final histologic report revealed presence of (i) one fibroblastic specimen maximal weight 100 g and (ii) two fibroblastic specimen’s maximal weight 50 g. (Figs 1 and 2.)

All specimens consisted of morphological and immunohistochemical characters compatible with leiomyoma not depicting signs of malignancy, developing local hydroponic degeneration *[SMA (+), Desmin (+), H-Caldesmon (+), C-Kit (−), DOG-1(−), CD34 (−), S100(−)].*

Patient discharged from hospital in stable condition on second day. She had uneventful postoperative course. She was followed 2 months later with transvaginal ultrasound, not revealing any residual myomas.

## DISCUSSION

Laparoscopy consists of gold standard procedure among many gynecologic procedures [8].

In cases of premenopausal patients focusing on fertility preservation, laparoscopic gynecologic interventions represent ultimate therapeutic mapping and proper treatment. This intervention is strongly correlated with decreased perioperative morbidity and increased postoperative recovery [9].

Undergoing subtotal or total laparoscopic hysterectomy, or in cases of laparoscopic myomectomy followed by morcellation, retained uterine fragments can infiltrate the peritoneal cavity and develop parasitic myomas.

Possible pathogenic mechanism represents the attachment of pedunculated subserous uterine leiomyomas to adjacent anatomic structures, losing their original uterine blood supply and receiving an auxiliary one [10].

Multiple foci of parasitic myomas inside the peritoneal cavity reflect the leiomyomatosis peritonealis disseminata (LPD) in women of reproductive age.

Parasitic myomas can be developed in any anatomic area of the peritoneal cavity. Most common areas are the peritoneum of the abdominal or pelvic wall, pouch of Douglas, omentum, colon and small intestines.

Van der Meulen *et al.* reported and analyzed six original cohort studies in order to evaluate the incidence of parasitic myomas after laparoscopic morcellation (Table 1).

**Table 1:** Van der Meulen et al: Parasitic myoma after laparoscopic morcellation: a systematic review of the literature. *BJOG* 2016;123:69–75

References	Study period (years)	LH (*n*)	LPSM (*n*)	LASH (*n*)	Follow-up	Parasitic myomas	Incidence (percentage with 95% CI)
Cuncinella *et al*.	3	102	321	–	–	4	Overall: 0.95 (0.32–2,25%) LPSM: 1.25 (0.42–2.96%)
Leren *et al*.	8	–	726	1744	–	3	0.12 (0.03–0.32%)^*^
Sinha *et al*.	8	–	505	–	7 days, 1 month, every 6 months (range 18-48 months)	1	0,20 (0.02–0,92%)^*^
Donnez *et al*.	17	–	–	1613	4–6 weeks, 1 year, every 2 weeks	9	0.56 (0.28–1.02%)
Ehdaivand *et al*.	5	352			–	2	0.57 (0.11–1.82%)

CI, confidence interval; LH, laparoscopic hysterectomy; LPSM, laparoscopic myomectomy; LASH, laparsocopic supravaginal hysterectomy; **–,** not provided.

^*^When not provided in the article, 95% CIs were calculated.

According to recent bibliography, first case of parasitic myoma after incomplete laparoscopic morcellation announced in 1997 by Ostrzenski.

Since then, possible options to decrease the recurrence risk of parasitic myomas are focusing on assiduous inspection of the peritoneal cavity after the completion of the surgical procedure, meticulous morcellation of the leiomyoma inside a contained endoscopic bag and performing mini laparoscopy.

Menderes *et al.* first described robotic dissection of parasitic leiomyoma diameter 3 × 3cm involving obturator fossa penetrating obturator foramen.

In cases with suspected malignancy, laparoscopic morcellation needs to be avoided. Therapeutic mapping focuses on exploratory laparotomy and histologic confirmation of all operative specimens.

## CONCLUSION

Parasitic myomas represent a rare entity with variable pathogenic origin. Proper and meticulous operative searching, using of laparoscopic bag during myomas morcellation, could solve such medical conditions.

## Disclosure of interest

All authors declare no financial interest with respect to this manuscript.
